# Inter-Reader Reliability of Early FDG-PET/CT Response Assessment Using the Deauville Scale after 2 Cycles of Intensive Chemotherapy (OEPA) in Hodgkin’s Lymphoma

**DOI:** 10.1371/journal.pone.0149072

**Published:** 2016-03-10

**Authors:** Regine Kluge, Lidia Chavdarova, Martha Hoffmann, Carsten Kobe, Bogdan Malkowski, Françoise Montravers, Lars Kurch, Thomas Georgi, Markus Dietlein, W. Hamish Wallace, Jonas Karlen, Ana Fernández-Teijeiro, Michaela Cepelova, Lorrain Wilson, Eva Bergstraesser, Osama Sabri, Christine Mauz-Körholz, Dieter Körholz, Dirk Hasenclever

**Affiliations:** 1 Department of Nuclear Medicine, University of Leipzig, Leipzig, Germany; 2 Clinic of Nuclear Medicine, National Hospital for Active Treatment in Oncology, Sofia, Bulgaria; 3 Department of Biomedical Imaging and Image-guided Therapy, Division of Nuclear Medicine, Medical University of Vienna, Vienna, Austria; 4 Department of Nuclear Medicine, University Hospital of Cologne, Cologne, Germany; 5 Dept. of PET and Molecular Imaging, Nicolaus Copernicus University, Collegium MedicumBydgoszcz, Poland; 6 Department of Nuclear Medicine, Hopital Tenon, Assistance Publique Hôpitaux de Paris, Faculté de médecine Pierre et Marie Curie, Paris, France; 7 Department of Paediatric Oncology, Royal Hospital for Sick Children, University of Edinburgh, Edinburgh, United Kingdom; 8 Karolinska University Hospital, Astrid Lindgrens Childrens Hospital, Stockholm, Sweden; 9 Pediatric Oncology Unit, Hospitales Universitarios Virgen Macarena y Virgen del Rocio, Sevilla, Spain; 10 Department of Pediatric Hematology and Oncology, University Hospital Motol and 2nd Medical Faculty of Charles University, Prague, Czech Republic; 11 Department of Nuclear Medicine, Blackrock Clinic, Dublin, Ireland; 12 Department of Paediatric Oncology, University Children’s Hospital Zurich, Switzerland; 13 Department of Pediatric Oncology, University of Halle, Halle/Saale, Germany; 14 Institute for Medical Informatics, Statistics and Epidemiology (IMISE), University of Leipzig, Leipzig, Germany; Northwestern University Feinberg School of Medicine, UNITED STATES

## Abstract

**Purpose:**

The five point Deauville (D) scale is widely used to assess interim PET metabolic response to chemotherapy in Hodgkin lymphoma (HL) patients. An International Validation Study reported good concordance among reviewers in ABVD treated advanced stage HL patients for the binary discrimination between score D1,2,3 and score D4,5. Inter-reader reliability of the whole scale is not well characterised.

**Methods:**

Five international expert readers scored 100 interim PET/CT scans from paediatric HL patients. Scans were acquired in 51 European hospitals after two courses of OEPA chemotherapy (according to the EuroNet-PHL-C1 study). Images were interpreted in direct comparison with staging PET/CTs.

**Results:**

The probability that two random readers concord on the five point D score of a random case is only 42% (global kappa = 0.24). Aggregating to a three point scale D1,2 vs. D3 vs. D4,5 improves concordance to 60% (kappa = 0.34). Concordance if one of two readers assigns a given score is 70% for score D1,2 only 36% for score D3 and 64% for D4,5. Concordance for the binary decisions D1,2 vs. D3,4,5 is 67% and 86% for D1,2,3 vs D4,5 (kappa = 0.36 resp. 0.56). If one reader assigns D1,2,3 concordance probability is 92%, but only 64% if D4,5 is called. Discrepancies occur mainly in mediastinum, neck and skeleton.

**Conclusion:**

Inter-reader reliability of the five point D-scale is poor in this interobserver analysis of paediatric patients who underwent OEPA. Inter-reader variability is maximal in cases assigned to D2 or D3. The binary distinction D1,2,3 versus D4,5 is the most reliable criterion for clinical decision making.

## Introduction

Several large treatment studies for Hodgkin lymphoma (HL) employ interim FDG-PET during chemotherapy to tailor treatment intensity–either by treatment intensification in non-adequate responders or by treatment reduction in adequate responders [1–5]. In general a binary decision has to be reached classifying a PET scan as adequate (= PET negative) or inadequate (= PET positive) metabolic response.

Several respective classification schemes have been developed in lymphoma during the last years [5–8]. In 2009 the Deauville 5-point scale was published as an international consensus for assessing metabolic response to chemotherapy [8]. The Deauville consensus eliminated the size criterion and introduced the liver as a second reference structure in addition to the mediastinum (D1 = no enhanced uptake, D2 = uptake < = mediastinum, D3 = uptake > mediastinum but < = liver, D4 = uptake moderately > liver, D5 = uptake strongly > liver). Current studies interpret either scores D3,4,5 (RAPID e.g.) or only scores D4,5 (RATHL e.g.) as PET positive. In 2012 the expert group agreed that in general scores D1,2,3 can be considered as complete metabolic response, so that mostly scores D4,5 should be classified as PET positive [9].

In current trials the interim PET scan is usually scored in a central reference reading by one or several expert groups. The conformity of the interpretation between experts using the same scales is a precondition for the comparability of results from different trials. Demonstrating excellent agreement among trained experts could be a first step towards handing over to a local reading process and a use of the method in clinical routine outside of studies. An International Validation Study reported good concordance among reviewers in ABVD (Doxorubicin, Bleomycin, Vinblastine, and Dacarbacine) treated advanced stage HL patients for the discrimination between score 1,2,3 and score 4,5 only. Inter-reader reliability of the full scale is not well characterised.

In addition, from real time review of images for the large European Network in Paediatric Hodgkin Lymphoma C1 study in classical HL (EuroNet-PHL-C1) starting in 2007 we had the impression that there is a substantial proportion of difficult to interpret cases after treatment with 2 cycles of OEPA (Vincristine, Etoposide, Prednisone and Doxorubicin) which is a more intensive chemotherapy than ABVD.

The aim of the present reference reading study was to evaluate the inter-observer agreement among experts using the D-scale after 2 cycles of intensive chemotherapy with OEPA.

In detail, the following questions should be answered:

1. How large is the inter-reader variability or the probability of concordance between different experts for exact Deauville categories or decisions on PET positivity/negativity using different thresholds of the scale?
2. Which are the main reasons for disagreement?
3. Is the inter-reader agreement compromised by low image quality or activated brown adipose tissue?

## Method

### Study population

This paper is based on FDG-PET/CT scans acquired at staging and after two cycles of OEPA chemotherapy of 100 randomly selected children and adolescents with classical HL in early (n = 40), intermediate (n = 21) or advanced (n = 39) stage. The early response PET/CT scan was scheduled between day 14 and day 17 after the last chemotherapy application in the second OEPA cycle, all staging PET/CT scans before introduction of chemotherapy. The patients are participants of the EuroNet-PHL-C1 trial and received initial staging and interim scans between 6/2009 and 2/2011 according to this trial (EudraCT 2006-000995-33). It was approved by the Ethical commission of University of Halle on Dec 13th 2006. All clinical investigation has been conducted according to the principles expressed in the Declaration of Helsinki. The participants of our study provided their informed written consent for the transfer of all imaging data and the storage and use of the anonymized data for scientific research. After consultation with the Ethical Commission of University of Leipzig an additional approval of sub-projects is not required if anonymized data are used.

The PET/CT scans were obtained in 51 trial sites in 9 European countries. The patients for this retrospective analysis were randomly selected irrespective of image quality, physiological uptake pattern like enhanced uptake in brown adipose tissue, use of i.v. contrast agent or technical specifications of the PET/CT scanner.

### PET/CT acquisition

The scans were obtained according to the local protocol of the individual PET centres but were in agreement with the EuroNet-PHL-C1 PET-manual and the Guidelines for ^18^F-FDG PET and PET-CT imaging in paediatric oncology of the European Association of Nuclear Medicine [10]. The administered ^18^F-FDG activity was 260±116 MBq, resp. 4.9±1.4 MBq/kg (mean ± SD). The interval between ^18^F-FDG injection and start of image acquisition was 73±20 min. Images were attenuation corrected using iterative reconstruction.

### Reference readers (R), allocation of PET/CT data sets and study template

The five R (MH Austria, CK and MD Germany (combined reading), BM Poland, FM France and LC Bulgaria) are nuclear medicine experts with special expertise in the interpretation of PET/CT in Hodgkin’s lymphoma. LC worked 3 years as R in the EuroNet PET/CT Core lab, FM, BM and MH received on-site training at the Core lab in Leipzig in the frames of the EAHC-funded Paediatric Hodgkin Network project, CK and MD are R in the PET/CT Core lab of the German Hodgkin lymphoma study group (HD 15–19 trials) and in long-term regular clinical contact with the EuroNet Core lab.

All PET/CT scans had been transferred in DICOM format via the Paediatric Hodgkin Network to a cloud server (TeleHERMES^TM^, HERMES Medical Solutions AB, Sweden) for real-time central review [11]. In the Core Lab in Leipzig the 200 PET/CT scans (baseline and interim PET/CT from 100 patients) were anonymised, provided with a new case number and saved in a special folder on the cloud server. No additional clinical information was added. The Rs were provided with right of access to the study folder on the Hermes cloud server, with a template for the recording of the evaluation results and a list to encode regions of tumour involvement (26 lymph node areas and 5 extra-nodal areas–spleen, liver, left and right lung, skeleton). All reviewers used the dedicated workstation of Hermes Medical Solutions, Sweden, for the evaluation of the PET/CT datasets. For the interpretation the software Hybrid Viewer was used allowing the interpretation of the interim PET/CT in direct comparison with the co-registered identical slices of the baseline PET/CT. The following parameters had to be filled in by the reviewers: scan quality (good– 0 points, limited– 1 point, poor– 2 points); activation of brown adipose tissue (no– 0 points, moderate– 1 point, strong– 2 points); artefacts (no or list of affected regions); suspicion for inflammatory lesions (region numbers); Deauville score and region number of the region with highest residual tumour uptake (in case of no residual uptake only the score D1 was filled in), and—if applicable–of the regions with second or third highest residual tumour uptake. Deauville scoring was strictly visual without supportive SUV measurements. In addition, comments could be saved in a free-text column.

The readers agreed before starting analysis of interim PET scans that new lesions (not present in the baseline PET) were not interpreted as lymphoma residuals if the patient was responding to treatment in initially involved sites and that only focal enhanced FDG uptake in initially involved areas was interpreted as residual tumour.

### Statistics

By design five nuclear physicians experienced in FDG-PET based response assessment in Hodgkin lymphoma independently scored the same 100 cases on the five category D scale. As the true D scores of the cases are not ascertainable we have focused on inter-rater agreement.

The most pertinent analysis answers a simple clinical question: If my primary nuclear physician scored my patient D score *d*–what is the chance that an independent second opinion assigns score *d* too? To answer this question we estimate the conditional probability of consensus within a random pair of readers who both look independently at the same random case and at least one of the readers assigns score *d*. Estimation follows Uebersax [12]. Note that by conditioning on at least one reader assigning score *d*, these estimates become less dependent on the underlying population (similar to sensitivity and specificity in the case of a diagnostic test and known gold standard). In addition we describe the overall probability of agreement of two randomly selected readers on a random case.

In the literature, inter-reader agreement is often analysed using kappa coefficients. Therefore we also include a kappa based analysis. We use Fleiss’s kappa (appropriate for multiple readers) in a version that is identical to Cohan’s kappa in the case of two readers. Note that conceptually kappa is not a measure of the degree of agreement, but measures degree of agreement beyond that expected based on the observed marginal score distributions of the rater [13]. Comparing kappa values across publications is problematic since kappa depends on the underlying population of cases [14].

The influence of image quality was investigated calculating measures of concordance for subgroups with good (0 quality point), slightly reduced (1 point) or reduced quality (> 1 point). The influence of FDG uptake in brown fat tissue was explored in a similar way.

## Results

For full transparency the individual scores of the five readers in the 100 cases are available as S1 Table.

### Differences in distribution of D scores

The distribution of assigned D scores varies among the different readers (Table 1). While readers R1, R2 and R4 detected no enhanced residual FDG uptake above background in about half of the patients, readers R3 and R5 found completely negative PET (D1) in only 10 or 14% of the cases, respectively. Interpretation of reader R5 shows a clear shift from D1 to D2 in comparison to the other readers, that of reader R3 from D1 and D2 to D3 and D4.

**Table 1:** Frequency of Deauville (D) scores by reader R1 –R5.

D score	R1	R2	R3	R4	R5
**1**	49	52	10	49	14
**2**	12	10	13	18	49
**3**	19	24	52	16	18
**4**	15	13	21	14	14
**5**	5	1	4	3	5

### Proportion of cases with consensus

An exact agreement among all five readers on an exact D score was reached in 12/100 cases (7x D1, 1 x D2, 0 x D3, 3 x D4, 1 x D5), an agreement of at least four of the readers in 29 cases (12 x D1, 2 x D2, 7 x D3, 6 x D4, 2 x D5). Since the decision D1 vs. D2 is of less clinical importance (always considered to be PET negative) we checked the agreement after summarizing D1 and D2 to one group. Under this condition an agreement among all 5 readers was reached in 22 cases (18 of them with score D1/2), four readers agreed in 58/100 cases (43 of them with score D1/2). An agreement of all five readers on a positive or negative PET using a threshold between D2 and D3 was reached in 33/100 cases (18 negative and 15 positive), an agreement of at least four of the readers in 71 cases (43 negative and 28 positive). Using a threshold between D3 and D4, all five readers agreed in 72 cases (65 negative and 7 positive), four readers agreed in 88 cases (76 negative and 12 positive).

### Examples of discordant cases

**Fig 1:**
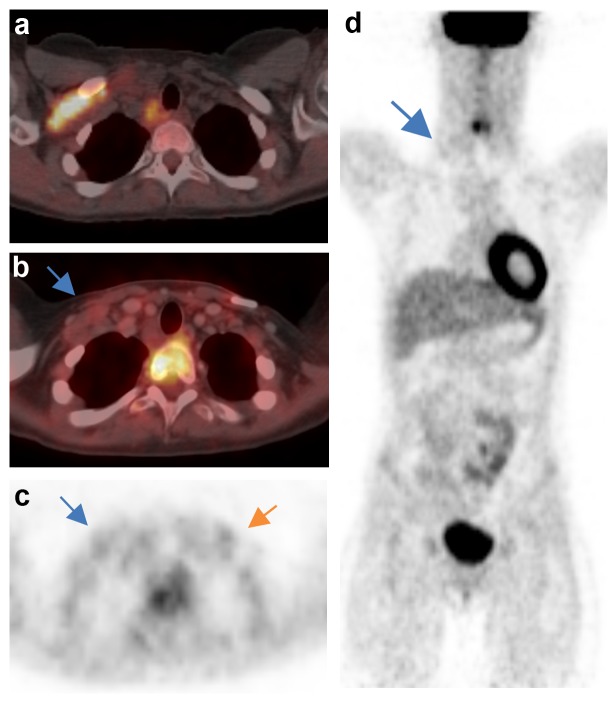
Discrepant D scoring of the right supraclavicular region. Case 63. a) Tumour involvement before treatment. b) Residual mass in this region after two cycles of OEPA. c) and d) slightly enhanced FDG uptake above background in this area. Interpretation might be complicated by a slightly enhanced FDG uptake symmetrically in the left supraclavicular area which, however, is in the surrounding of a catheter and by the inhomogeneous uptake in the mediastinum. Deauville score of the readers R1-5, respectively: 1-1-3-1-2. Courtesy Dept. Nuclear Medicine, Karolinska University Hospital, Stockholm, Sweden.

In Fig 1 the different use of D1 among the reviewers is exemplified.

**Fig 2:**
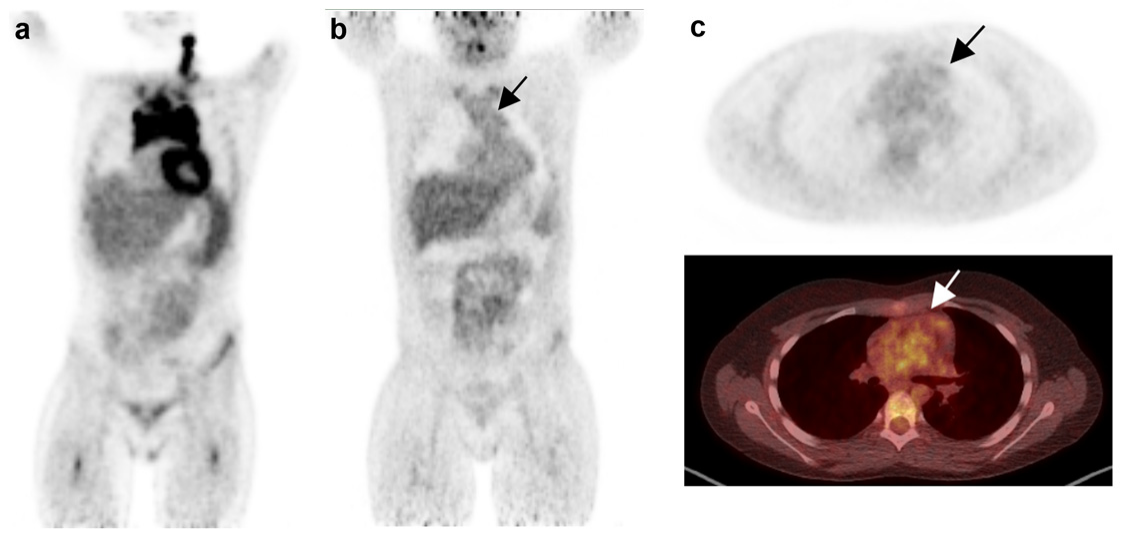
Residual uptake in the mediastinum vs. thymus. Case 12: a) Lymphoma involvement of the upper and middle mediastinum including the thymus before treatment. b) and c) very slightly enhanced uptake as compared to the mediastinum after two cycles ChT. The interpretation might be complicated by the difficult decision whether this finding implies residual tumor uptake or physiological but inhomogeneous uptake in the thymus. Deauville score of the readers R1-5, respectively: 1-1-3-3-3. Courtesy Dept. Nuclear Medicine, Blackrock Clinic, Dublin, Ireland.

**Fig 3:**
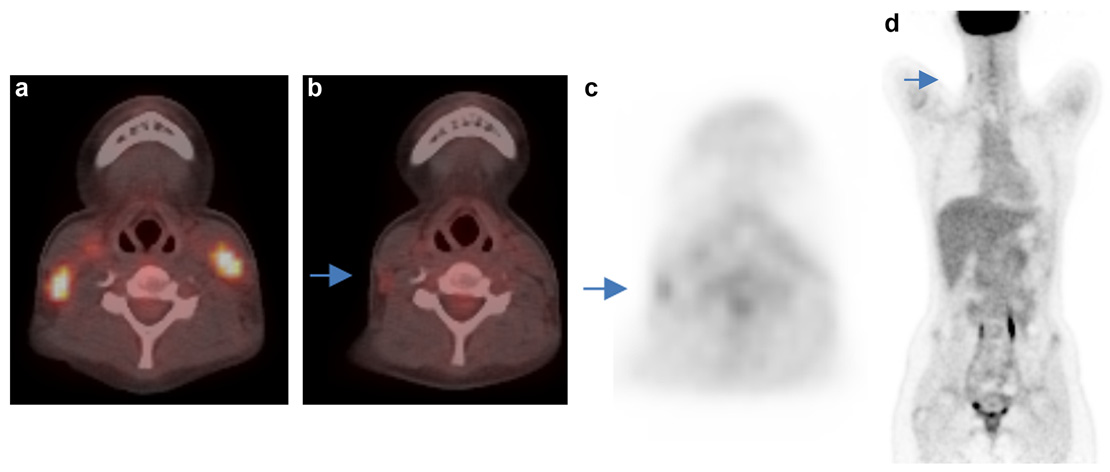
Discrepant D scoring of the neck region. Case 44: a) Lymphoma involvement at both sides of the neck before treatment. b) and c) residual lymph node on the right neck with slightly enhanced FDG uptake after to cycles ChT. d) The residual uptake is less than in the liver, but it is difficult to decide if it is above or below the MBP uptake. Deauville score of the readers R1-5, respectively: 2-3-3-1-3. Courtesy Dept. Clinical Physiology, Sahlgrenska University Hospital, Gothenburg Sweden.

Typical problems in the interpretation D1,2 vs. D3,4,5 are demonstrated in Figs 2 and 3.

**Fig 4:**
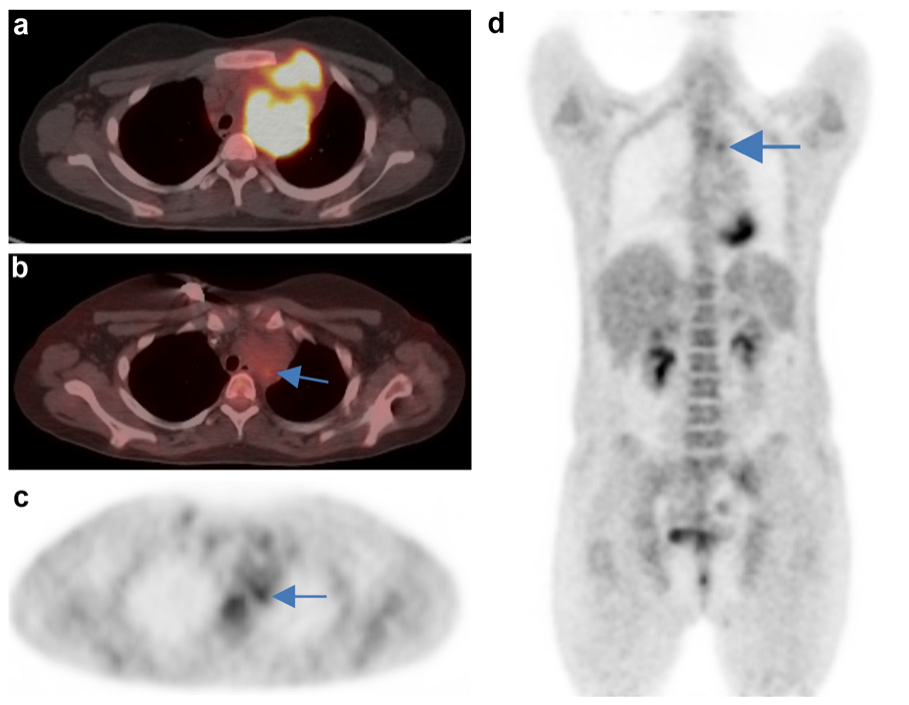
Discrepant D scoring of the mediastinum. Case 39: a) extensive lymphoma involvement of the left side of the upper mediastinum before treatment. b) residual mass after two cycles ChT. c) highest residual FDG uptake in the dorsal part of the residuum. D) It is difficult to decide if this highest FDG uptake is above or below the liver uptake. The interpretation is complicated by the small size of the hottest part of the residuum. Deauville score of the readers R1-5, respectively: 3-3-4-4-4. Courtesy M. Reinhardt, Dept. Nuclear Medicine, Pius-Hospital, Oldenburg, Germany.

**Fig 5:**
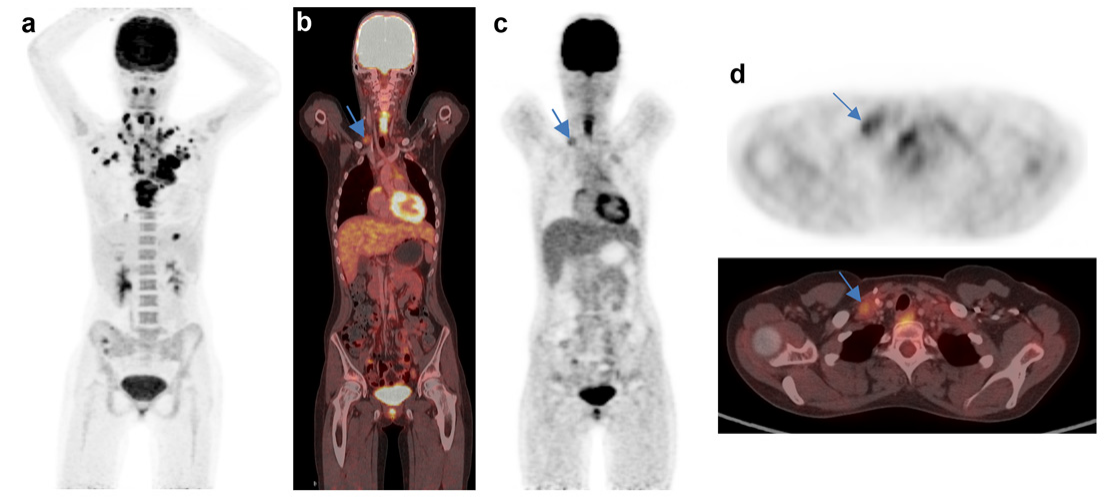
Discrepant D scoring of the right supraclavicular region. Case 2.: a) extensive lymphoma involvement including the right supraclavicular region before treatment. b—d) enhanced FDG uptake in a right supraclavicular node clearly exceeding that of the mediastinum. It is difficult to decide if the nodal uptake is higher or less than the liver uptake. Deauville score of the readers R1-5 for the supraclavicular region, respectively: 3-4-3-3-4. Courtesy Dept. Nuclear Medicine, University Hospital Erlangen, Germany.

Figs 4 and 5 illustrate typical problems in the interpretation D1,2,3 vs. D4,5.

### Measures of agreement

Table 2 summarizes measures of agreement for the full five point D-scale as well as for derived three and two category aggregated scales. For each scale we provide estimates of the overall probability of agreement of two randomly selected readers on a random case. Also estimates of the category- specific conditional probability of agreement given that at least one in a pair of readers scores a certain category are provided. In addition, global kappa coefficients and the range of the ten pair-wise kappa coefficients among the five readers are reported. For pair-wise kappa coefficients between readers see S2 Table.

**Table 2:** Agreement statistics for Deauville (D) scoring among five readers.

	Overall probability of concordance	Specific probability of concordance for categories					Global kappa	Range of pairwise kappas
Scale		D1	D2	D3	D4	D5		
**Five categories: D1, D2, D3, D4, D5**	0.422	0.520	0.255	0.360	0.494	0.556	0.244	0.144–0.387
**Three categories: D1,2, D3, D4,5**	0.604	0.705		0.360	0.642		0.344	0.222–0.485
**Two categories: D1,2, D3,4,5**	0.674	0.705		0.636			0.358	0.189–0.514
**Two categories: D1,2,3, D4,5**	0.864	0.916			0.642		0.559	0.428–0.710

The probability that two random readers assign exactly the same D score is only 42% overall. Specific probabilities of concordance are highest for the extreme values D1 or D5 (> 50%) and lowest for D2 (25%) and D3 (36%). Aggregation of the scale to only three categories (D1,2 vs. D3 vs. D4,5) increases the overall probability of concordance to 60%. However, the probability to agree on score D3 remains low (36%). The restriction of the scale to a binary decision (PET positive or negative) results in a further increase in probability to concord, with markedly better results when the threshold is made between D3 and D4 (86%) as compared to a threshold between D2 and D3 (67%). The concordance rate in the latter case profits from the large fraction of negative cases (D1,2,3: 92%), while the probability of concordance on a positive case (D4,5) is only 64%.

Kappa for agreement on exactly the same D score is globally only 0.24 ranging from 0.14 to 0.39 in pairs of reviewers. Limiting the interpretation to a binary decision D1,2 “negative” versus D345 “positive” improves the global kappa to 0.36. Pairwise kappa values range between 0.19 and 0.51. This broad range is mainly due to the systematically higher rate of D3 in reader R3. The Kappa values of the four other readers range between 0.39 and 0.51. Using a binary decision D1,2,3 versus D4,5 leads to a global kappa 0.56 indicating a moderate to good concordance.

### Concordance of interpretation in different regions of the body

In order to explore whether there are regions with particular problems we analysed discrepancy patterns by region (Table 3) taking the frequency of initial involvement into account. To eliminate regional discrepancies which are caused by different interpretation of region borders, the 21 lymph node areas were aggregated to 8 larger lymph node regions: upper, lower neck and supraclavicular to “neck” (left and right), infraclavicular and axillar to “axilla” (left and right), nodes in upper, middle, lower mediastinum, both sides lung hilus and recessus phrenicocostalis to “mediastinum”, upper and lower paraaortic nodes and nodes at spleen and liver hilus and intestinum to “abdomen”, iliac and inguinal nodes to “pelvic” (left and right). In addition, 5 extranodal regions were evaluated: left and right lung, spleen, liver and skeleton. We used the three category D1,2 vs. D3 vs D4,5 scale in order to focus on clinically relevant decisions.

**Table 3:** Discrepancy analysis by region using the D1,2 vs D3 vs D4,5 three category scale.

	Patientwise reader comparisons N = 100				Pairwise reader comparisons N = 1000			
	Initially involved regions	All reader concord	Only minor discrepancies	Major discrepancies	Number of concordant pairs	Number of minor discrepancies	Number of major discrepancies	% major among pairwise discrepancies
**Mediastinum**	88	40	52	8	694	288	18	5,9
**Neck left**	74	72	18	10	849	120	31	20,5
**Neck right**	73	84	7	9	908	56	36	39,1
**Abdomen**	27	96	2	2	982	8	10	55,6
**Skeleton**	7	93	2	5	970	8	22	73,3
**Axilla left**	32	97	2	1	986	10	4	
**Axilla right**	24	98	1	1	989	8	3	
**Spleen**	18	99	1	0	996	4	0	
**Lung right**	14	100	0	0	1000	0	0	
**Lung left**	12	98	1	1	990	4	6	
**Pelvic left**	10	99	1	0	996	4	0	
**Pelvic right**	4	100	0	0	1000	0	0	
**Liver**	1	100	0	0	1000	0	0	

Discrepancies between D1,2 and D4,5 are considered major. Discrepancies involving adjacent categories (D1,2 and D3 or D3 and D4,5) are called minor.

Many discrepancies occurred in the mediastinum which was initially involved in 88 cases and on which consensus of all readers was achieved in 40/100 cases only. 288 of 306 (94%) pairwise reader discrepancies differed only by one category suggesting grading ambiguity.

The left neck was involved in 74 patients. Unanimous reader consensus was reached in 72 of 100 cases. Among the 1000 pairwise reader comparisons 849 were concordant. Among the 151 pairwise reader discrepancies 31 (21%) were major (i.e. clearly negative D1,2 versus clearly positive D4,5).

The right neck was involved in 73 patients. Unanimous reader consensus was reached in 84 of 100 cases. Among the 1000 pairwise reader comparisons 908 of 1000 were concordant. Among the 92 pairwise reader discrepancies 36 (39%) were major.The skeleton was involved in only seven patients, but this region stands out with the highest proportion of major reader discrepancies (22 major and 8 minor pairwise discrepancies).

In the other nine regions including axillary regions, spleen, abdomen and lung unanimous reader consensus rate was above 95%. These regions were typically negative in the interim PET. In these regions neither scoring nor interpretation/detection discrepancies played a relevant role.

### Influence of image quality or brown adipose tissue (BAT) uptake

Good quality was attested by all five readers to 58 of the interim PET/CT. For 25 scans only one of the readers mentioned reduced quality, for 17 scans reduced quality was stated by more than one reader. The probability to concord on the D score was not different in cases with adequate or reduced quality (“good quality” 0.610 vs. “limited” one reader 0.595 vs. “limited” several readers 0.618; Uebersax method). Enhanced FDG-uptake in BAT was stated by at least one of the readers in 17 cases, in 11 cases the majority of the readers mentioned enhanced BAT uptake. The probability to concord on the D score decreased slightly with the occurrence of BAT uptake (0.611 without BAT uptake vs. 0.571 “BAT” one reader vs. 0.500 “BAT” three or more readers).

## Discussion

In our study five readers with particular experience in the interpretation of PET scans in HL independently scored 100 cases randomly selected from a large clinical trial. All readers were able to directly compare interim PET/CT with the respective staging PET/CT. Inter-reader reliability of the 5 point D scale was rather poor. The estimated probability of two random readers to concord on the exact same D-score in a random case was only 42%. In particular there is massive inter-reader variability in cases assigned for D2 or D3.

Aggregating to a three point scale (D1,2 vs D3 vs D4,5) or to a binary scale (D1,2 vs D3,4,5) only marginally improved the probability of concordance (60% and 67% respectively).

Only the distinction D 1,2,3 versus D4,5 may be sufficiently reliable for clinical decision making (86% probability of agreement). This good result is driven by excellent concordance on negative cases while the conditional probability of concordance if one reader scores D4,5 was only 64%.

### Possible explanations for the observed discrepancies

#### Ambiguity in interpreting reference uptake levels

The distribution of score values varied markedly between readers (Table 1). This suggests that different interpretations of the score level definition used in the Deauville scoring may play a role.

This is especially relevant for the background definition (D1 vs. D2). The physiological uptake pattern causes broad variability of local backgrounds. Obviously some readers consequently use the local background (any visible uptake in a tumour residual is per definition >D1) while others also grade lesions with a slightly enhanced uptake in a near-zero background into group D1 (see Fig 1).

Another source of discrepancies is the definition of the mediastinal region (D2 vs. D3). While earlier scoring systems used the term “mediastinal blood pool” [6] the D scoring system uses the term “mediastinum”[8]. The inhomogeneity of the FDG-uptake in this anatomically multifarious region increases the risk of discrepancy. This problem is less relevant in the liver (D3 vs. D4) because of the large size of the organ and the only moderate inhomogeneity of the physiological FDG uptake, especially in children.

Finally, for the classification D4 vs. D5 the criterion “markedly enhanced uptake” is prone to subjective interpretation.

#### Ambiguity in assessing residual tumour uptake and comparing grey levels

The D criteria do not precisely prescribe which part of the residual uptake is to be used for comparison with reference regions. Is it the mean uptake in the whole residual uptake area or the mean uptake of its hottest part (how delineated?) or the maximum uptake which should be used for the visual comparison?

Inter-reader differences may also occur during the process of the visual comparison of the residual uptake with the reference regions. It can be difficult to compare grey levels across a distance in particular in case of small residuals (see Figs 2 or 4). In some cases residuum and reference structures cannot be compared directly since they are located in different planes. In addition, the comparison may be compromised by optical illusion effects like the simultaneous brightness contrast illusion or the subjective colour constancy (see Edward Adelson Chequer-shadow illusion 1995) [15].

Our recently published semi-automatic qPET quantification method [16] uses the average of the maximum voxel with the three highest adjacent voxels to quantify the residual uptake and a standardised VOI to determine the reference liver signal in an attempt to eliminate these sources of variability.

#### Enrichment of borderline cases

Concordance rates between readers and kappa coefficients depend on the proportion of clear-cut cases in the sample. In our data in 68/100 cases at least one reader scored residual uptake as D3 indicating a high proportion of borderline cases. This preponderance of borderline cases is typical in our setting (16) and particularly impairs the discrimination D2 vs. D3. This may be due to using intensive OEPA chemotherapy; after ABVD the distinction between metabolically responding and non-responding cases may be more clear-cut (see below).

#### Problem regions

Mainly three regions gave rise to discrepant interpretations: mediastinum, skeleton and neck lymph nodes (compare Table 3). In more than 60% of the cases readers differed in the D score of the mediastinum. Mostly minor discrepancies occurred concerning D1,2 vs D3 or less often D3 vs. D4. Here most of the sources of variability discussed above collude: a high proportion of borderline uptake in the residual lymphoma mass, inhomogeneous FDG uptake in the reference region “mediastinum”, differences among readers in the way of looking on it and the discrimination of physiological structures like vessel walls or thymus from typically weak residual uptake.

Large discrepancies in the interpretation occurred in the skeleton. In nearly two thirds of the cases in whom at least one of the readers detected an active tumour residuum in the skeleton, these findings were either misinterpreted or overlooked by other readers. The risk of overlooking a residual uptake is increased in the skeleton because of the large size of the region and the low frequency of involvement.

The interpretation of the neck region is complicated due to difficulties in discriminating lymph nodes from salivary glands or other structures with physiologically enhanced FDG uptake, often small size of the residuum and large distance to the reference organs. In addition, the distinction between original lymphoma and newly appeared inflammatory lymph nodes may be intricate.

#### Acquisition variability, quality and brown fat

Many acquisition parameters may influence the image characteristics and the quality of the FDG PET scans [17–18]. This study evaluated 100 cases whose images were acquired in 51 different PET/CT centres in nine countries for a large European clinical trial with substantial acquisition variability. However, limited scan quality as reported by the readers was not associated with increased discrepancy rates indicating that acquisition variability is not the main cause for the observed discrepancies.

Strong FDG-uptake in brown adipose tissue (BAT) is well-known to complicate the interpretation of FDG-PET/CT scans. This is a particular issue in the post-treatment situation when small residuals have to be identified or excluded. Discrepancy rates were slightly higher in scans with high FDG uptake in BAT. However, the inter-reader agreement remained poor even in good quality scans without BAT uptake.

### Comparison with other inter reader studies using Deauville criteria

Although the D scale is widely considered as well established most validation papers only report concordance data on the binary discrimination D1,2,3 versus D4,5. Only Oki at al. [19] report concordance data concerning the full five point scale after 2 ABVD cycles. Their results (pairwise kappa values: 0.13, 0.29, and 0.38) are in agreement with our own (0.14–0.39).

As in our study the literature consistently shows that concordance is highest for the binary distinction D1,2,3 versus D4,5. After 2 ABVD several groups report pairwise kappa values in the order of 0.8: Biggi (0.69–0.84)[20], Oki (0.78–0.91)[19], and Barrington (0.74–0.96)[21]. These results compare favourably with our results after 2 OEPA (0.43–0.71). Furth et al [22] report a kappa value of 0.75 in a small series after 2 OEPA, but they excluded “unclear” D3 cases. The recalculated kappa value for the whole patient group is 0.68. Horning 2010 [23] report kappa 0.50 after 3 R-CHOP in DLBCL patients. Furth et al. [24] report lesion-wise kappa values in paediatric NHL; recalculation of patient wise kappa values yield kappa = 0.41. Assessing D1,2,3 versus D45 appears more clear-cut after 2 ABVD compared to other chemotherapy.

Inter reader concordance for the distinction D1,2,3 versus D45 depends on the proportion of border line cases in the respective population. In our data (after 2 OEPA), in 68% of the cases D3 was assigned by at least one reader; 24% of the cases can be considered D3 (i.e. the mean of the scores rounds to 3). This D3 proportion is remarkably higher than reported by Barrington (6/50; 12%) and Oki (6/229; 2.4%) after 2 ABVD.

OEPA is a more intense chemotherapy regimen than ABVD. Thus uptake in incomplete metabolic responders may be shifted from higher residual uptake intensities towards the D34 border. However the proportion of positive cases is not lower in our data than in the data of Oki or Biggi [19,20]. This on the other hand suggests a shift of clearly negative cases towards the D34 border, e.g. due to granulocyte activation or an enhanced debriding reaction.

### Limitations

Inter-reader concordance of interim PET scoring may depend on the population included and possibly on the nature and cycle number of previously administered chemotherapy. As our results were obtained in a pediatric patient population we cannot exclude that physiological uptake in the thymus and activation of brown adipose tissue marginally increased the rate of discrepancies. We used a particular intensive chemotherapy (OEPA), which may have increased the proportion of difficult borderline cases as compared to less intense regimen (e.g. ABVD). Our images are from a multicenter setting; monocentric images may be easier to interpret.

### Conclusion

We have shown that inter-reader reliability of the complete 5 point D scale is poor in a real world study setting. In particular there is significant inter-reader variability in cases assigned for D 2 or 3. The distinction D 1,2,3 versus 4,5 is the most reliable criterion for clinical decision making. The use of a semi-automatic algorithm for comparison of the residual uptake intensity with the reference levels (16) may help to avoid inter-reader discordances and make decisions on treatment reduction or–intensification in PET-guided trials more reliable.

## Supporting Information

S1 Table Raw data: Individual D-scores of 100 cases by 5 readers. (DOCX)

S2 Table Agreement between pairs of reviewers. Cohen’s Kappa values for the precise Deauville score, for the binary decision D1,2 vs. D3,4,5 or for the binary decision D1,2,3 vs. D4,5.
(DOCX)
