# Supplementary material for: Inter-Reader Reliability of Early FDG-PET/CT Response Assessment Using the Deauville Scale after 2 Cycles of Intensive Chemotherapy (OEPA) in Hodgkin’s Lymphoma
Source: PLoS One. 2016 Mar 10;11(3):e0149072. doi: 10.1371/journal.pone.0149072 (PMC4786307; doi:10.1371/journal.pone.0149072)
Supplement: S1 Table — (DOCX) [file pone.0149072.s001.docx]

**Table S1**: **Raw data: Individual D-scores of 100 cases by 5 readers**

|  | Deauville Scores | | | | |
| --- | --- | --- | --- | --- | --- |
| caseNr | Reader1 | Reader2 | Reader3 | Reader4 | Reader5 |
| 1 | 1 | 1 | 2 | 1 | 2 |
| 2 | 5 | 4 | 5 | 5 | 5 |
| 3 | 5 | 5 | 5 | 5 | 5 |
| 4 | 1 | 1 | 3 | 2 | 2 |
| 5 | 4 | 3 | 4 | 4 | 4 |
| 6 | 2 | 1 | 3 | 1 | 2 |
| 7 | 5 | 4 | 4 | 3 | 4 |
| 8 | 3 | 1 | 3 | 2 | 1 |
| 9 | 3 | 3 | 3 | 1 | 2 |
| 10 | 3 | 3 | 4 | 3 | 2 |
| 11 | 1 | 1 | 3 | 1 | 2 |
| 12 | 1 | 1 | 3 | 3 | 3 |
| 13 | 1 | 1 | 3 | 1 | 2 |
| 14 | 3 | 1 | 3 | 3 | 4 |
| 15 | 3 | 1 | 3 | 1 | 2 |
| 16 | 1 | 1 | 2 | 1 | 2 |
| 17 | 1 | 1 | 2 | 2 | 1 |
| 18 | 1 | 1 | 3 | 2 | 3 |
| 19 | 4 | 4 | 4 | 4 | 4 |
| 20 | 1 | 1 | 1 | 1 | 2 |
| 21 | 2 | 3 | 3 | 2 | 2 |
| 22 | 4 | 1 | 3 | 4 | 2 |
| 23 | 1 | 1 | 3 | 1 | 2 |
| 24 | 3 | 2 | 3 | 2 | 3 |
| 25 | 1 | 1 | 1 | 1 | 1 |
| 26 | 2 | 2 | 3 | 2 | 3 |
| 27 | 1 | 1 | 3 | 1 | 2 |
| 28 | 3 | 1 | 3 | 3 | 3 |
| 29 | 1 | 1 | 1 | 1 | 1 |
| 30 | 1 | 2 | 3 | 1 | 3 |
| 31 | 2 | 2 | 2 | 2 | 2 |
| 32 | 1 | 4 | 4 | 4 | 4 |
| 33 | 1 | 1 | 4 | 1 | 2 |
| 34 | 1 | 1 | 3 | 1 | 2 |
| 35 | 2 | 1 | 1 | 4 | 2 |
| 36 | 1 | 1 | 2 | 1 | 1 |
| 37 | 1 | 1 | 2 | 1 | 3 |
| 38 | 2 | 3 | 3 | 2 | 2 |
| 39 | 3 | 3 | 4 | 4 | 4 |
| 40 | 4 | 1 | 4 | 2 | 3 |
| 41 | 1 | 1 | 1 | 1 | 1 |
| 42 | 3 | 4 | 4 | 2 | 3 |
| 43 | 2 | 1 | 3 | 1 | 2 |
| 44 | 2 | 3 | 3 | 1 | 3 |
| 45 | 1 | 1 | 2 | 1 | 1 |
| 46 | 1 | 1 | 3 | 1 | 2 |
| 47 | 1 | 3 | 4 | 1 | 4 |
| 48 | 1 | 1 | 3 | 2 | 1 |
| 49 | 3 | 3 | 3 | 2 | 3 |
| 50 | 2 | 1 | 3 | 1 | 2 |
| 51 | 3 | 3 | 3 | 2 | 2 |
| 52 | 1 | 1 | 1 | 1 | 1 |
| 53 | 3 | 1 | 3 | 1 | 3 |
| 54 | 2 | 2 | 3 | 3 | 2 |
| 55 | 1 | 2 | 3 | 1 | 2 |
| 56 | 4 | 3 | 4 | 4 | 4 |
| 57 | 1 | 1 | 2 | 1 | 1 |
| 58 | 4 | 3 | 4 | 1 | 4 |
| 59 | 1 | 1 | 2 | 1 | 2 |
| 60 | 2 | 2 | 3 | 2 | 2 |
| 61 | 3 | 1 | 3 | 1 | 2 |
| 62 | 1 | 3 | 3 | 1 | 2 |
| 63 | 1 | 1 | 3 | 1 | 2 |
| 64 | 5 | 4 | 4 | 5 | 5 |
| 65 | 1 | 3 | 4 | 1 | 2 |
| 66 | 1 | 1 | 2 | 1 | 2 |
| 67 | 4 | 4 | 5 | 4 | 5 |
| 68 | 3 | 4 | 3 | 3 | 3 |
| 69 | 4 | 2 | 4 | 4 | 2 |
| 70 | 1 | 3 | 2 | 2 | 2 |
| 71 | 1 | 1 | 1 | 1 | 1 |
| 72 | 1 | 3 | 3 | 3 | 3 |
| 73 | 1 | 1 | 3 | 1 | 2 |
| 74 | 3 | 1 | 3 | 1 | 2 |
| 75 | 1 | 1 | 3 | 3 | 2 |
| 76 | 1 | 2 | 3 | 1 | 2 |
| 77 | 1 | 1 | 1 | 1 | 1 |
| 78 | 4 | 3 | 3 | 3 | 2 |
| 79 | 1 | 1 | 2 | 1 | 2 |
| 80 | 2 | 1 | 4 | 4 | 2 |
| 81 | 1 | 1 | 3 | 1 | 2 |
| 82 | 3 | 3 | 4 | 3 | 3 |
| 83 | 3 | 3 | 3 | 3 | 2 |
| 84 | 1 | 3 | 3 | 4 | 2 |
| 85 | 1 | 4 | 5 | 1 | 4 |
| 86 | 3 | 3 | 3 | 4 | 2 |
| 87 | 4 | 3 | 3 | 1 | 4 |
| 88 | 1 | 1 | 3 | 1 | 2 |
| 89 | 5 | 3 | 4 | 3 | 4 |
| 90 | 1 | 2 | 3 | 2 | 2 |
| 91 | 1 | 1 | 1 | 1 | 1 |
| 92 | 1 | 1 | 2 | 3 | 2 |
| 93 | 3 | 3 | 3 | 2 | 3 |
| 94 | 4 | 4 | 4 | 3 | 5 |
| 95 | 1 | 1 | 3 | 3 | 3 |
| 96 | 4 | 1 | 3 | 1 | 3 |
| 97 | 4 | 4 | 4 | 4 | 4 |
| 98 | 4 | 4 | 4 | 4 | 4 |
| 99 | 1 | 1 | 3 | 1 | 1 |
| 100 | 4 | 4 | 1 | 1 | 2 |
