# Supplementary material for: Inter-Reader Reliability of Early FDG-PET/CT Response Assessment Using the Deauville Scale after 2 Cycles of Intensive Chemotherapy (OEPA) in Hodgkin’s Lymphoma
Source: PLoS One. 2016 Mar 10;11(3):e0149072. doi: 10.1371/journal.pone.0149072 (PMC4786307; doi:10.1371/journal.pone.0149072)
Supplement: S2 Table — Cohen’s Kappa values for the precise Deauville score, for the binary decision D1,2 vs. D3,4,5 or for the binary decision D1,2,3 vs. D4,5. (DOCX) [file pone.0149072.s002.docx]

**Table S2**: **Agreement between pairs of reviewers.**

| **Deauville 5 point scale** | | | | | | **Global kappa** |
| --- | --- | --- | --- | --- | --- | --- |
|  | **R1** | **R2** | **R3** | **R4** | **R5** | 0.244 |
| **R1** | 1.000 | 0.356 | 0.191 | 0.387 | 0.276 |  |
| **R2** | 0.356 | 1.000 | 0.144 | 0.314 | 0.179 |  |
| **R3** | 0.191 | 0.144 | 1.000 | 0.177 | 0.285 |  |
| **R4** | 0.387 | 0.314 | 0.177 | 1.000 | 0.181 |  |
| **R5** | 0.276 | 0.179 | 0.285 | 0.181 | 1.000 |  |
|  | | | | | |  |
| **Deauville three categories 1/2 versus 3 versus 4/5** | | | | | | 0.344 |
|  | **R1** | **R2** | **R3** | **R4** | **R5** |  |
| **R1** | 1.000 | 0.435 | 0.325 | 0.393 | 0.485 |  |
| **R2** | 0.435 | 1.000 | 0.240 | 0.330 | 0.370 |  |
| **R3** | 0.325 | 0.240 | 1.000 | 0.222 | 0.342 |  |
| **R4** | 0.393 | 0.330 | 0.222 | 1.000 | 0.381 |  |
| **R5** | 0.485 | 0.370 | 0.342 | 0.381 | 1.000 |  |
|  |  |  |  |  |  |  |
| **Deauville two categories 1/2 versus 3/4/ 5** | | | | | |  |
|  | **R1** | **R2** | **R3** | **R4** | **R5** | 0.358 |
| **R1** | 1.000 | 0.514 | 0.285 | 0.438 | 0.491 |  |
| **R2** | 0.514 | 1.000 | 0.239 | 0.412 | 0.467 |  |
| **R3** | 0.285 | 0.239 | 1.000 | 0.189 | 0.263 |  |
| **R4** | 0.438 | 0.412 | 0.189 | 1.000 | 0.386 |  |
| **R5** | 0.491 | 0.467 | 0.263 | 0.386 | 1.000 |  |
|  |  |  |  |  |  |  |
| **Deauville two categories 1/2/3 versus 4/5** | | | | | | 0.559 |
|  | **R1** | **R2** | **R3** | **R4** | **R5** |  |
| **R1** | 1.000 | 0.507 | 0.571 | 0.503 | 0.650 |  |
| **R2** | 0.507 | 1.000 | 0.531 | 0.428 | 0.603 |  |
| **R3** | 0.571 | 0.531 | 1.000 | 0.522 | 0.710 |  |
| **R4** | 0.503 | 0.428 | 0.522 | 1.000 | 0.526 |  |
| **R5** | 0.650 | 0.603 | 0.710 | 0.526 | 1.000 |  |

Cohen’s Kappa values for the precise Deauville score, for the binary decision D1,2 vs. D3,4,5 and for the binary decision D1,2,3 vs. D4,5.
